# Combinations of posaconazole and tacrolimus are effective against infections with azole-resistant *Aspergillus fumigatus*

**DOI:** 10.3389/fcimb.2025.1550457

**Published:** 2025-04-25

**Authors:** Eliane Vanhoffelen, Tine Van Win, Eva Van Braeckel, Agustin Reséndiz-Sharpe, Bruno P. A. Cammue, Katrien Lagrou, Karin Thevissen, Greetje Vande Velde

**Affiliations:** ^1^ Department of Imaging and Pathology, Biomedical MRI Unit, KU Leuven, Leuven, Belgium; ^2^ Department of Respiratory Medicine, Ghent University Hospital, Ghent, Belgium; ^3^ Department of Internal Medicine and Pediatrics, Respiratory Infection and Defense Lab (RIDL), Faculty of Medicine and Health Sciences, Ghent University, Ghent, Belgium; ^4^ Department of Microbial and Molecular Systems (M²S), Microbial and Plant Genetics (CMPG), KU Leuven, Leuven, Belgium; ^5^ Leuven Plant Institute, KU Leuven, Leuven, Belgium; ^6^ Department of Microbiology, Immunology and Transplantation, Laboratory of Clinical Microbiology, KU Leuven, Leuven, Belgium; ^7^ Department of Laboratory Medicine, National Reference Center for Mycosis, University Hospitals Leuven, Leuven, Belgium

**Keywords:** posaconazole, tacrolimus, FK506, Transplantation, synergy, *Aspergillus fumigatus*, azole-resistance, biofilm

## Abstract

**Background:**

Solid organ transplant recipients on immunosuppressants such as tacrolimus are at increased risk of developing pulmonary aspergillosis, a severe to deadly complication with limited treatment options, especially against azole-resistant strains. This study investigates the antifungal interaction between posaconazole and tacrolimus, prompted by a case where a liver transplant recipient on tacrolimus experienced unexpected eradication of chronic *Aspergillus fumigatus* colonization following posaconazole prophylaxis.

**Methods:**

We compared the combined antifungal activity of posaconazole and tacrolimus against azole-sensitive and resistant *A. fumigatus in vitro* against planktonic isolates and biofilm formation and *in vivo* in *Galleria mellonella* larvae, to evaluate the potential benefit over posaconazole monotherapy.

**Results:**

The posaconazole-tacrolimus combination demonstrated a 4-fold increase in efficacy against azole-resistant isolates and a 30-fold increase against an azole-sensitive strain, in contrast to voriconazole. Moreover, this combination enhanced antifungal activity by 4- to 15-fold against biofilm formation of azole-sensitive strains, though no synergy was observed against azole-resistant biofilms. *In vivo* studies in *Galleria mellonella* confirmed a 2- to 7-fold decrease in fungal burden of both azole-sensitive and azole-resistant strains when combining posaconazole with tacrolimus, relative to posaconazole alone.

**Conclusion:**

*In vitro* and *in vivo* findings confirm that posaconazole may offer therapeutic benefits for treating *A. fumigatus* infections in patients receiving tacrolimus. These results warrant further confirmation in mice and exploration of their clinical implications.

## Graphical Abstract

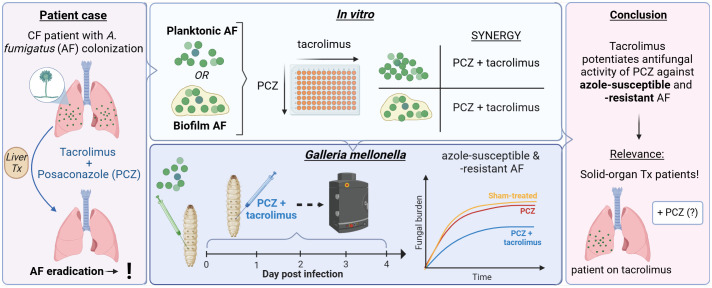

## Introduction

Aspergillosis, predominantly caused by the environmental fungus *Aspergillus fumigatus*, is the most frequent pulmonary mold infection. Inhalation of airborne *A. fumigatus* conidia can cause a wide spectrum of respiratory diseases leading to significant morbidity and mortality, with invasive pulmonary aspergillosis (IPA) being the most serious presentation (Denning, 2024; Janssens et al., 2024). Treatment options for *A. fumigatus* infections are limited to three drug classes – triazoles, polyenes and echinocandins – of which the use can be complicated by drug-drug interactions, lack of oral formulations and adverse effects (Lamoth and Calandra, 2022). The European ESCMID-ECMM-ERS guidelines recommend voriconazole (VCZ) and isavuconazole as first-line treatment for IPA, though posaconazole (PCZ) is non-inferior to VCZ (Ullmann et al., 2018; Maertens et al., 2021). However, the emergence of azole resistance is a major concern in the management of *A. fumigatus* infections, with a prevalence of 7.1% in Belgium (Resendiz-Sharpe et al., 2021). The most common mutations in triazole-resistant *A. fumigatus* isolates are TR_34_/L98H and TR_46_/Y121F/T289A in the *cyp51A* gene, typically due to environmental selection by the extensive use of fungicides in agriculture (Resendiz-Sharpe et al., 2021). Triazole-resistant *A. fumigatus* infections have higher mortality rates compared to those infected with triazole-susceptible isolates, with studies reporting 47-88% mortality in triazole-resistant IPA (Lestrade et al., 2019; WHO fungal priority pathogens list to guide research, development and public health action, 2022). Additionally, treatment of aspergillosis can be further compromised by biofilm formation, consisting of aggregated cells surrounded by a protective extracellular matrix that protects the fungus from antifungals and host responses, making biofilms very difficult to eradicate (Kernien et al., 2018). Taken together, there is an urgent need for novel treatment strategies against *A. fumigatus* infections, as highlighted by the WHO fungal priority pathogen list (WHO fungal priority pathogens list to guide research, development and public health action, 2022).

Solid organ transplant (SOT) recipients are an important group of immunocompromised patients for whom IPA is a serious complication with high rates of graft loss and a 3-month mortality rate of 15-90% (Neofytos et al., 2021; Young et al., 2024). Treatment of IPA in SOT recipients is complex due to frequent drug-drug interactions and organ-specific toxicity. VCZ and isavuconazole remain the recommended treatment, with inhaled amphotericin B, echinocandins and VCZ preferred for prophylaxis, though no comparative studies of antifungal therapy or prophylaxis have been performed in this population (Ullmann et al., 2018).

This study was triggered by a case of a cystic fibrosis (CF) patient with chronic *A. fumigatus* colonization who underwent liver transplantation. He received tacrolimus-based immunosuppression alongside PCZ prophylaxis to prevent the development of IPA in the immediate post-transplant setting. Surprisingly, respiratory cultures remained negative post-transplant (**Supplementary Data**: Case study). This unexpected clearance of *A. fumigatus* from native CF lungs after 14 years of chronic colonization immediately post-liver transplant raised the question whether tacrolimus potentiated PCZ’s antifungal activity. Given the potential relevance of this question regarding antifungal choices in the SOT population, we systematically investigated whether tacrolimus or other calcineurin inhibitors such as rapamycin can increase PCZ’s antifungal activity against azole-susceptible and resistant *A. fumigatus.* We also included VCZ in this study as VCZ is the standard of care for both treatment and prophylaxis of IPA in this patient group. We tested for *in vitro* synergy against planktonic cultures and biofilm formation, and *in vivo* potentiation in a *Galleria mellonella* aspergillosis model.

## Materials and methods

### Strains and chemicals

The *Aspergillus fumigatus* strains used in the *in vitro* assays were azole-sensitive [CBS117202; Westerdijk Institute, Netherlands (Nouripour-Sisakht et al., 2017)] and azole-resistant (ASFU5318 and ASFU3216; Prof Katrien Lagrou, UZ Leuven, Belgium) clinical isolates. CBS117202 originates from broncho-alveolar lavage, ASFU5318 was isolated from sputum of a mucoviscidosis patient and ASFU3216 from sputum of a hematology patient. Isolates were not obtained from the case study. Both azole-resistant isolates harbor modifications in the *cyp51A* gene and its promotor; ASFU5318 has mutation TR_34_/L98H, whereas ASFU3216 has mutation TR_46_/Y121F/T289A. These mutations were confirmed by sequencing as previously described (Vermeulen et al., 2015). Azole-resistance was initially determined using the European Committee on Antimicrobial Susceptibility Testing (EUCAST) broth microdilution reference method for filamentous fungi and resistance clinical breakpoints for *A. fumigatus* (EUCAST, 2020; Breakpoint tables for interpretation of MICs for antifungal agents, version 10.0, 2020). No resistance against amphotericin B was found in any of the isolates. Stock solutions (200x) of rapamycin (Bio-connect, Huissen, Netherlands), tacrolimus (Sigma-Aldrich, St. Louis, US) and posaconazole (TCI Europe, Zwijndrecht, Belgium) were prepared in dimethyl sulfoxide (DMSO) (VWR International, Belgium).

For *in vivo* experiments, we used previously validated bioluminescent *A. fumigatus* strains expressing a codon-optimized red-shifted firefly luciferase: triazole-susceptible [wild type WT)] (Af_luc_OPT_red__WT) and triazole-resistant (TR_34_/L98H) (Af_luc_OPT_red__TR_34_) (Resendiz-Sharpe et al., 2022). The minimum inhibitory concentrations (MICs) of posaconazole for the bioluminescent WT and TR_34_/L98H strains are 0.125 mg/L and 0.5 mg/L, respectively (Resendiz-Sharpe et al., 2022). *A. fumigatus* conidial suspensions for *G. mellonella* infection were prepared as previously described (Vanhoffelen et al., 2023).

### *In vitro* susceptibility testing

Preparation of *A. fumigatus* spore suspensions, dilution of antifungal agents, and determination of MIC values of antifungal agents, immunosuppressants and their combinations followed the CLSI M38-A2 standard protocol with an additional spectrophotometric readout to quantify fungal growth inhibition (> 90%) (Clinical and Laboratory Standards Institute (CLSI) and Wayne, 2008). Immunosuppressant concentrations were selected as the highest concentration without intrinsic antifungal activity. Since we used the CBS117202 strain as a quality control in the CLSI M38-A2 standard protocol (2008), the reported MIC values should only be interpreted for the identification of synergistic combinations, and not for external comparison with regulatory or diagnostic cutoffs. MIC values are means of at least four independent experiments. Based on these MICs, the fractional inhibitory concentration index (FICI) was calculated to identify synergistic combinations using the equation ∑FICI = FICI_A_ + FICI_B_ = (C_A_/MIC_A_) + (C_B_/MIC_B_), where MIC_A_ and MIC_B_ are the MICs of compound A and B alone, and C_A_ and C_B_ are their concentrations in combination. Combinations were considered as synergistic if FICI ≤ 0.5, additive if 0.5 < FICI ≤ 1.0, indifferent if 1.0 < FICI ≤ 4.0 and antagonistic if FICI > 4.0 (Odds, 2003; Meletiadis et al., 2010).

### *In vitro* biofilm inhibition assay

*A. fumigatus* spores (5.10^4^/mL) were incubated statically at 37°C in RPMI-1640 buffered with MOPS for pH 7 (Sigma Aldrich) in round-bottomed 96-well microtiter plates (TPP, Tradingen, Switzerland) for 48h in the presence of antifungals (final DMSO concentration of 1%). Biofilm biomass was quantified using the viability dye XTT after washing the biofilms with PBS to remove non-adherent cells. To this end, 110 μL of a XTT solution (0.25 mg/mL in PBS, 10 μM menadione; Sigma Aldrich, St Louis, USA) was added to the washed biofilms. After 2h at 37°C, the absorption (OD_490 nm_) of 100 μL of the converted XTT solution was measured. The Biofilm Inhibitory Concentration (BIC) value was then determined as the minimal concentration of the compound that completely inhibited biofilm formation, i.e. resulting in less than 20% of the (spectrophotometric) read-out of the control treatment (1% DSMO). BIC values are means of at least three independent experiments.

### *Galleria mellonella* aspergillosis model

**Figure 1:**
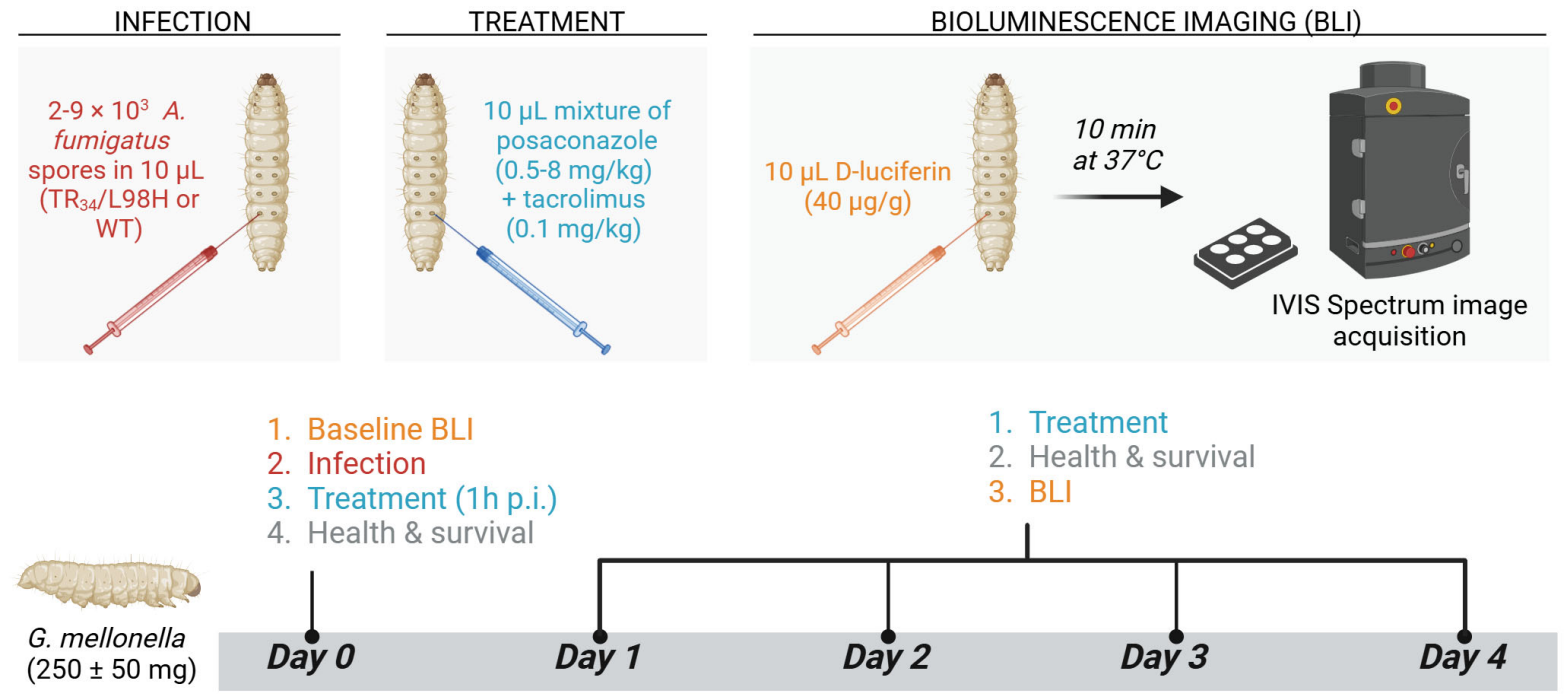
*Galleria mellonella* aspergillosis model for *in vivo* evaluation of PCZ potentiation by tacrolimus. In-house bred, healthy *G. mellonella* larvae of 250 ± 50 mg (n = 10 per group) were housed individually in 12-well plates (Cellstar®, Greiner bio-one, Austria) without food and stored in the dark at 37°C during the experiment. On day 0, *A. fumigatus* inocula ranging between 2000 (TR_34_/L98H) and 9000 (WT) conidia per 10 µL were injected intra-hemocoel via the last right proleg using a Hamilton® syringe (25 µl, 702SN, 30 G, Switzerland). Non-infected control groups received 10 µL PBS. Posaconazole (PCZ) (Noxafil, MSD, USA) and tacrolimus (Prograf, Astellas Pharma Inc, Japan) solutions (IV formulations) were diluted in 0.9% sterile saline to achieve doses of 8, 4, 2, 1 or 0.5 mg/kg for PCZ and 0.1 mg/kg for tacrolimus. Treatments were freshly prepared and administered daily, starting from 1 h post infection, by intra-hemocoel injection in alternately the last left and right prolegs. Infected sham-treated control groups received 0.9% saline, and non-infected control groups received the combination of PCZ and tacrolimus to assess treatment toxicity. Larvae were followed-up daily for four days post infection (p.i.) by *in vivo* BLI, survival and health scoring (based on movement, melanization and survival). For *in vivo* BLI, larvae were injected intra-hemocoel with 10 µL D-luciferin (40 µg/g in PBS) 10 min before image acquisition (Vanhoffelen et al., 2023). Figure created with BioRender.

*In vivo* potentiation of posaconazole (PCZ) by tacrolimus was tested in a *G. mellonella* model of azole-resistant and sensitive *A. fumigatus* infection as shown and explained in **Figure 1**. To non-invasively quantify fungal burden over time, daily bioluminescence imaging (BLI) of live larvae was performed over four days post infection using an IVIS Spectrum imaging system (Revvity, USA), as previously optimized (Vanhoffelen et al., 2023; Vanhoffelen et al., 2024).

### Statistical analysis

All statistical analyses were performed using GraphPad Prism version 8.0.2 (GraphPad Software, USA). Longitudinal health scores and log_10_-transformed *in vivo* BLI data were analyzed using two-way ANOVA or mixed effect analyses, applying the Geisser-Greenhouse correction for sphericity. Tukey’s multiple comparison test was used to compare the main treatment effects over time. Larvae that died during the experiment were handled as missing values in all BLI and health score statistical analysis by fitting a mixed model rather than by repeated measures ANOVA. However, to avoid visual survival bias (i.e., the apparent decline in mean group value when larvae with the highest fungal burden succumb to infection), especially important in the context of treatment experiments, we carried forward the last measurement before death to the missing next timepoints in the graphical representations only. To calculate the overall decrease in fungal burden compared to the sham-treated group over the course of an experiment, the longitudinal *in vivo* BLI fluxes were first normalized to the sham-treated control by dividing every single datapoint by the corresponding daily average of the sham-treated group, obtaining values <1 when fungal burden is lower and >1 when fungal burden is higher than the sham-treated average. After log_10_-transformation, the area under the curve (AUC) of these normalized values was calculated with baseline y=0 (because log_10_[1]=0), representing the amount of reduction in fungal burden compared to the sham-treated group. These AUC’s were then compared between groups by one-way ANOVA with Sidak’s multiple comparison correction. Mantel-Cox log-rank testing was performed for survival analysis. *P* values of <0.05 were considered significant.

## Results

### Tacrolimus and rapamycin act synergistically with posaconazole but not voriconazole against planktonic *A. fumigatus* cultures

We determined minimal inhibitory concentrations (MIC) of PCZ, VCZ and immunosuppressants tacrolimus and rapamycin alone and in combination against one azole-sensitive *A. fumigatus* isolate (CBS117202) and two azole-resistant *A. fumigatus* isolates (ASFU5318 and ASFU3216) (**Table 1**). Both azole-resistant isolates harbor modifications in the *cyp51A* gene and its promotor; *A. fumigatus* isolate ASFU5318 has mutation TR_34_/L98H, whereas ASFU3216 has mutation TR_46_/Y121F/T289A. The MIC of both immunosuppressants was > 8 µg/mL against all isolates.

**Table 1:** *In vitro* synergy between posaconazole (PCZ), voriconazole (VCZ) and immunosuppressants tacrolimus or rapamycin against *A. fumigatus* planktonic cultures.

Strain	MIC (µg/mL)^a^ *[FICI]*					
	No immunosuppressant		TACROLIMUS		RAPAMYCIN	
	PCZ	VCZ	PCZ	VCZ	PCZ	VCZ
CBS117202	0.5	0.5	0.015 *[0.03]*	0.25 *[0.53]*	0.015 *[0.03]*	0.25 *[0.53]*
ASFU3216	2	16	0.5 *[0.31]*	8 *[1.5]*	0.5 *[0.27]*	16 *[1.5]*
ASFU5318	1	8	0.25 *[0.28]*	4 *[1]*	0.25 *[0.26]*	8 *[1.25]*

a MIC (minimal inhibitory concentration) values are means of at least four independent biological experiments and determined according to the CLSI M38-A2 protocol and spectrophotometric readout > 90%. MIC values should be interpreted for synergistic combination identification only. FICI values for each combination are shown between brackets. Synergistic activity with FICI ≤ 0.5 is indicated in grey. Tacrolimus and rapamycin concentration was 2 µg/ml in all combinations (MIC tacrolimus or rapamycin alone was >8 µg/ml), except for the combination with PCZ against ASFU3216 and ASFU5318, where rapamycin concentration was 8 µg/ml (MIC rapamycin alone was >32 µg/ml). PCZ, posaconazole; VCZ, voriconazole.

We found that the interaction between both immunosuppressants and PCZ qualified as synergistic against all tested *A. fumigatus* isolates (FICI < 0.5 for 2 µg/mL of the immunosuppressants or 8 µg/mL rapamycin against the resistant isolates as indicated). In general, PCZ activity could be increased by 4-fold in combination with tacrolimus or rapamycin against azole-resistant isolates, and by 30-fold against the azole-sensitive isolate. Hence, these data demonstrate a synergistic interaction between PCZ and tacrolimus or rapamycin, that is independent of most common azole-resistance mechanisms reported for *A. fumigatus*. On the other hand, the interaction between both immunosuppressants and VCZ qualified as indifferent against all of the tested strains.

### Tacrolimus and rapamycin act synergistically with posaconazole against *A. fumigatus* biofilms

The combination of VCZ or PCZ with immunosuppressants was also tested against biofilm formation by the same azole-sensitive and resistant *A. fumigatus* isolates (**Table 2**). The biofilm minimal inhibitory concentration (BIC) of both immunosuppressants was > 8 µg/mL against all isolates. For the azole-sensitive *A. fumigatus* isolate, the interaction between both immunosuppressants and PCZ, and between rapamycin and VCZ qualified as synergistic, based on FICI calculations. In these setups, azole activity could be increased by 4- to 15-fold in combination with the immunosuppressants. However, these synergies could not be observed in biofilm inhibitory setups using azole-resistant *A. fumigatus* isolates.

**Table 2:** *In vitro* synergy between posaconazole (PCZ), voriconazole (VCZ) and immunosuppressants tacrolimus or rapamycin against *A. fumigatus* biofilm formation.

Strain	BIC (µg/mL)^a^ *[FICI]*					
	No immunosuppressant		TACROLIMUS		RAPAMYCIN	
	PCZ	VCZ	PCZ	VCZ	PCZ	VCZ
CBS117202	0.125	0.5	0.03 *[0.24]*	0.25 *[0.53]*	0.008 *[0.07]*	0.06 *[0.13]*
ASFU3216	2	16	1 *[0.63]*	16 *[3]*	2 *[1.25]*	16 *[3]*
ASFU5318	0.5	4	0.5 *[1.06]*	2 *[0.75]*	0.5 *[1.06]*	4 *[1.5]*

a BIC (biofilm minimal inhibitory concentration) values are means of at least three independent biological experiments. Read-out was based on metabolic activity of biofilm cells (Van Dijck et al., 2018); BIC was determined as the minimal concentration resulting in < 20% viability after viability staining. FICI values for each combination are shown between brackets. Synergistic activity with FICI ≤ 0.5 is indicated in grey. Tacrolimus and rapamycin concentration was 2 µg/ml in all combinations (BIC tacrolimus or rapamycin alone was >8 µg/ml against all isolates). PCZ, posaconazole; VCZ, voriconazole.

### Tacrolimus potentiates antifungal effect of posaconazole against azole-sensitive *A. fumigatus* in *G. mellonella*

In the light of the case study and given the few *in vivo* reports on azole combination treatment with tacrolimus (Chen et al., 2013; Lewis et al., 2013; Lee et al., 2018; Zhong et al., 2018), we aimed to investigate whether our *in vitro* findings of synergy between tacrolimus and PCZ against *A. fumigatus* isolates can be translated to an *in vivo G. mellonella* infection model. Tacrolimus was dosed at 0.1 mg/kg, which falls within the therapeutically relevant intravenous range for human SOT recipients (Astellas Pharma, ; Staatz and Tett, 2004).

**Figure 2:**
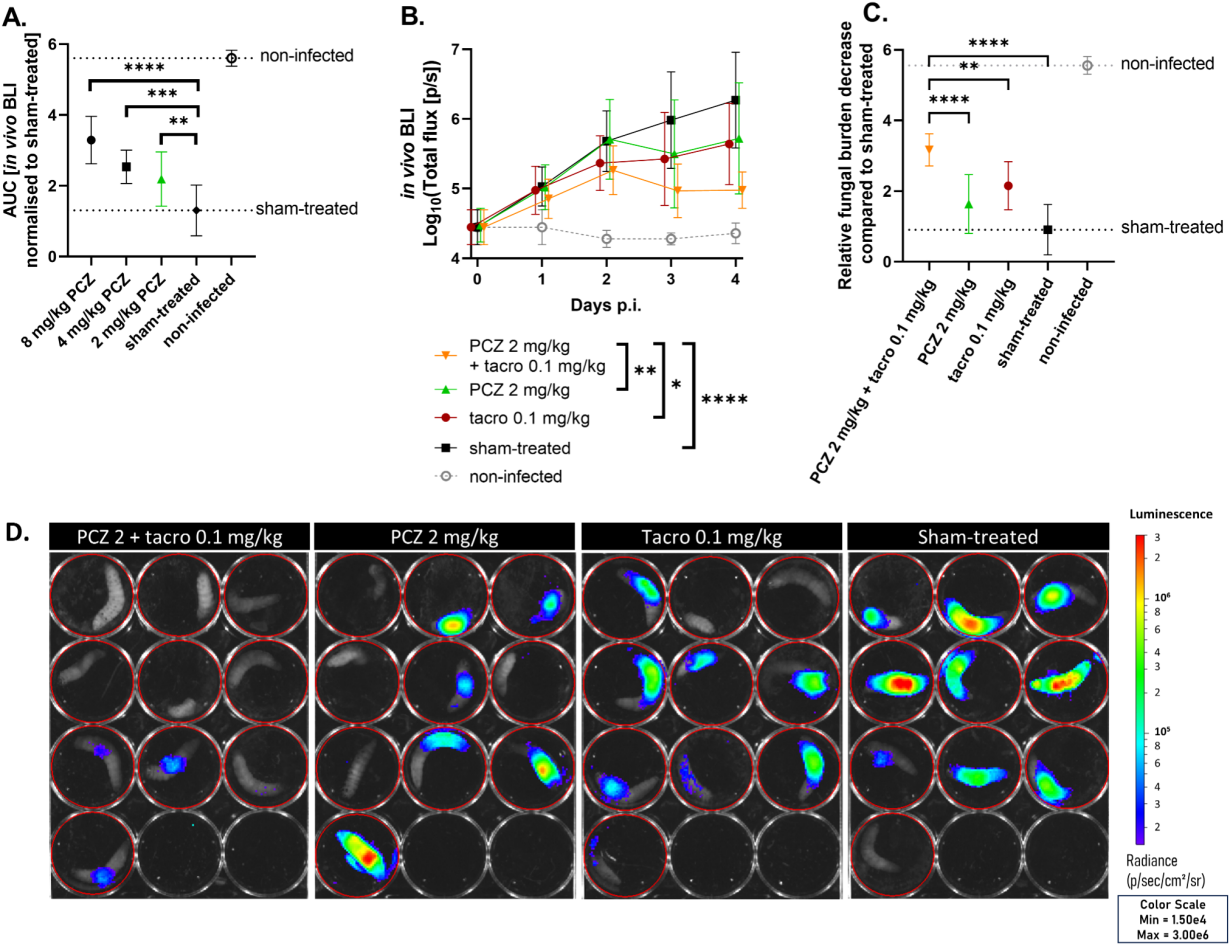
Tacrolimus potentiates PCZ antifungal efficacy *in vivo* against azole-sensitive *A. fumigatus* in *Galleria mellonella.* **(A)** The relative reduction in fungal burden of different PCZ doses compared to sham-treated larvae against an azole-sensitive *A*. *fumigatus* infection in *G*. *mellonella*, measured as area under the curve of normalized *in vivo* BLI signal over 4 days post infection (p.i.). **(B)** *In vivo* BLI signals in larvae infected with azole-sensitive *A*. *fumigatus*, comparing the effect of PCZ, tacrolimus or the combination of both on fungal burden over time. Graphs show means ± standard deviations (SD) (*n* = 10). **(C)** Overall fungal burden reduction relative to sham-treated larvae, measured as area under the curve of panel **(B)** after normalization for the sham-treated group. **(D)** Visual representation of *in vivo* BLI signal in infected larvae on day 4 p.i. BLI, bioluminescence imaging; [p/s], photons per second; PCZ, posaconazole; Tacro, tacrolimus. *P < 0.05; **P < 0.01; ***P <0.001; ****P < 0.0001.

First, we titrated therapeutically relevant PCZ doses against an azole-sensitive *A. fumigatus* infection in *G. mellonella* (MIC = 0.125 mg/L). Larvae were infected with bioluminescent *A. fumigatus*, allowing to non-invasively quantify the *in vivo* fungal burden in each larva over time via the bioluminescent signal (**Supplementary Figure S1**) (Vanhoffelen et al., 2023). Based on the overall efficacy of PCZ observed over four days, we selected the lowest significant dose (2 mg/kg) for combination tests with tacrolimus (**Figure 2A**). The combination of daily PCZ treatment at 2 mg/kg with daily tacrolimus at 0.1 mg/kg significantly reduced larval fungal burden over time compared to both single compounds or sham-treated larvae (**Figures 2B, D**). To assess the overall benefit compared to sham-treated larvae, we normalized the longitudinal BLI signals of all larvae to the sham-treated group’s average at each timepoint and calculated the AUC, thus representing the relative overall reduction in fungal burden compared to the sham-treated group over the course of the experiment (**Figure 2C**). This revealed a 2-fold greater reduction in fungal burden for the combination of 2 mg/kg PCZ and tacrolimus over PCZ alone, comparable to 8 mg/kg PCZ monotherapy (**Figure 2A**). Moreover, larvae receiving the combination were significantly healthier than sham-treated larvae, reflecting lower fungal burden (**Supplementary Figures S3A, B**). Non-infected larvae treated with tacrolimus and PCZ showed no significant decline in health compared to sham-treated controls, confirming the combination’s tolerability in *G. mellonella*. Overall, these findings support the translatability of the *in vitro* synergy between PCZ and tacrolimus to an *in vivo* model of azole-sensitive *A. fumigatus*.

### Tacrolimus potentiates antifungal effect of posaconazole against azole-resistant *A. fumigatus* in *G. mellonella*

**Figure 3:**
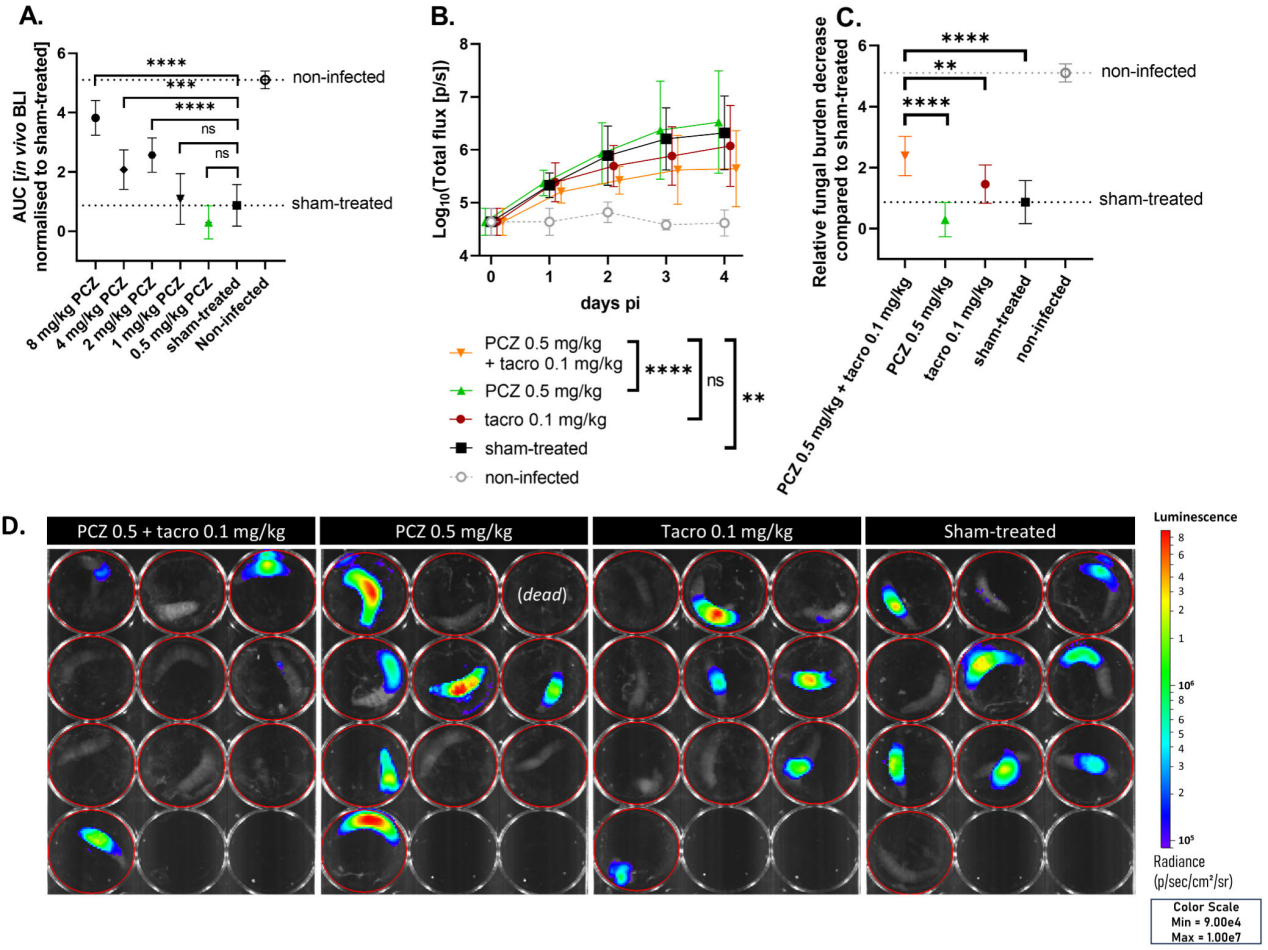
Tacrolimus potentiates PCZ antifungal efficacy *in vivo* against azole-resistant *A*. *fumigatus* in *Galleria mellonella.* **(A)** The relative reduction in fungal burden of different PCZ doses compared to sham-treated larvae against an azole-resistant (TR_34_/L98H) *A*. *fumigatus* infection in *G*. *mellonella*, measured as area under the curve of normalized *in vivo* BLI signal over 4 days post infection (p.i.). **(B)** *In vivo* BLI signals in larvae infected with azole-resistant *A*. *fumigatus*, comparing the effect of PCZ, tacrolimus or the combination of both on fungal burden over time. Graphs show means ± standard deviations (SD) (*n* = 10). **(C)** Overall fungal burden reduction relative to sham-treated larvae, measured as area under the curve of panel **(B)** after normalization for the sham-treated group. **(D)** Visual representation of *in vivo* BLI signal in infected larvae on day 4 p.i. BLI, bioluminescence imaging; [p/s], photons per second; PCZ, posaconazole. Tacro: tacrolimus. **P < 0.01; ***P <0.001; ****P < 0.0001; ns, non-significant.

Likewise, we titrated PCZ against an azole-resistant (TR_34_/L98H) *A. fumigatus* infection in *G. mellonella* to identify a subtherapeutic PCZ dose for this resistant strain (MIC = 0.5 mg/L) (**Supplementary Figure S2**). This allowed us to test whether tacrolimus potentiation of PCZ could counteract the resistance against PCZ monotherapy. Larvae received daily PCZ doses (0.5-8 mg/kg) for four days post infection. Based on the relative reduction in fungal burden compared to sham-treated larvae, we selected 0.5 mg/kg PCZ for combination tests with tacrolimus, as this was the only dose that showed no antifungal effect in any of the larvae (**Figure 3A**). Combining this subeffective PCZ dose with 0.1 mg/kg of tacrolimus significantly reduced the *in vivo* fungal burden compared to either treatment alone, thus overcoming fungal resistance to PCZ monotherapy. Neither PCZ (0.5 mg/kg) nor tacrolimus (0.1 mg/kg) significantly reduced the fungal burden when administered alone (**Figures 3B, D**). When combined with tacrolimus, PCZ reduced the overall fungal burden approximately 7-fold more than when used alone (**Figure 3C**), achieving levels similar to those observed with 2 to 4 mg/kg PCZ monotherapy (**Figure 3A**). No significant differences in survival or health were observed between groups (**Supplementary Figures S3C, D**). In conclusion, we confirmed the *in vivo* potentiation of PCZ by tacrolimus against azole-resistant (TR_34_/L98H) *A. fumigatus* in *G. mellonella.*

## Discussion

Based on our clinical case suggesting a positive interaction between PCZ and tacrolimus, we investigated the *in vitro* effects of PCZ or VCZ with tacrolimus and rapamycin against *A. fumigatus*. Synergy was observed between PCZ and both tacrolimus and rapamycin against planktonic *A. fumigatus*, increasing antifungal activity 4-fold against azole-resistant *A. fumigatus* isolates and 30-fold against an azole-sensitive isolate. Against biofilm formation of the azole-sensitive isolate, PCZ activity increased 4- to 15-fold in combination with tacrolimus or rapamycin, while no synergy was observed against azole-resistant biofilms. To our knowledge, this is the first report on antifungal synergy between PCZ and tacrolimus against *A. fumigatus.*

The interaction between both immunosuppressants and VCZ was indifferent against all tested planktonic cultures, consistent with previous studies (Steinbach et al., 2004b; Steinbach et al., 2004a; Lamoth et al., 2013). In a biofilm inhibition setting, VCZ synergized with rapamycin but not tacrolimus. In contrast, Gao L. et al. reported synergy between VCZ and tacrolimus against 6 out of 10 A*. fumigatus* biofilms, while PCZ showed overall indifference (Gao and Sun, 2015). These discrepancies may result from different tacrolimus concentrations or from isolate-specific synergies. Isavuconazole was also reported to show synergy with tacrolimus against 4 out of 10 azole-sensitive and resistant *A. fumigatus* isolates (Schwarz and Dannaoui, 2020). Moreover, *in vitro* synergies between azoles and tacrolimus or rapamycin were shown against multiple other fungal species, including azole-sensitive and resistant *Candida* spp (Sun et al., 2008; Denardi et al., 2015; Tome et al., 2018).*, Mucorales* spp. (Narreddy et al., 2010; Shirazi and Kontoyiannis, 2013)*, E. dermatitidis* (Gao et al., 2017), *Sporothrix* spp. (Borba-Santos et al., 2017) and *C. neoformans* (Del Poeta et al., 2000).

To confirm the synergistic effect in a simple *in vivo* setting, we tested the antifungal efficacy of PCZ and tacrolimus in *G. mellonella* larvae. Potentiation between clinically relevant doses of tacrolimus and PCZ was confirmed, with 2- and 7-fold reductions in fungal burden of azole-sensitive and -resistant *A. fumigatus* isolates, respectively, compared to PCZ alone. We used BLI for non-invasive, longitudinal fungal burden quantification of bioluminescent *A. fumigatus* in *G. mellonella*, as BLI was shown to have a higher dynamic range than colony-forming units and greater sensitivity than health and survival readouts in this model (Vanhoffelen et al., 2023). The latter was also confirmed in this study. Moreover, BLI directly measures the impact of treatment on fungal burden, whereas health and survival readouts reflect downstream effects of disease.

While no studies have investigated the combination of PCZ and tacrolimus against *A. fumigatus* in mice, a few reports suggest potential interactions between these agents against other fungal pathogens (Chen et al., 2013; Lewis et al., 2013; Lee et al., 2018; Zhong et al., 2018). In one study, combining tacrolimus (0.5 mg/kg i.p.) with PCZ improved outcomes in mice infected with azole-susceptible *C. albicans*, but no synergy was observed against azole-resistant strains (Chen et al., 2013). In a rat catheter model of fluconazole-resistant *C. albicans* biofilms on the other hand, combination of fluconazole and tacrolimus completely inhibited biofilm formation (Uppuluri et al., 2008). Also in a non-lethal murine model of cutaneous mucormycosis, combining tacrolimus (1 mg/kg i.p.) with PCZ significantly reduced lesions and *Rhizopus oryzae* fungal burden (Lewis et al., 2013). In the context of *A. fumigatus*, the combination of VCZ with topical tacrolimus demonstrated superior efficacy against *A. fumigatus*-induced keratitis, reducing inflammatory cytokine expression and improving clinical scores compared to VCZ only groups (Zhong et al., 2018).

In patients, tacrolimus is mainly used for rejection prophylaxis in SOT recipients. Because of this immunosuppression, IPA is one of the most common invasive fungal infections in this population, especially in lung transplant recipients but also in heart, kidney and liver recipients (Singh and Husain, 2013; Neofytos et al., 2021). Consequently, primary antifungal prophylaxis is often administered to lung transplant recipients or other SOT recipients with additional risk factors for IPA (Ullmann et al., 2018). There is a lack of large studies comparing optimal antifungal prophylaxis and treatment for IPA in SOT recipients, but generally, inhaled amphotericin B, echinocandins and VCZ are used as prophylaxis while VCZ, isavuconazole and liposomal amphotericin B are preferred for treatment (Ullmann et al., 2018; Neofytos et al., 2021). PCZ has been used in the setting of lung transplantation in CF patients on tacrolimus (Berge et al., 2009). However, caution is needed when co-administering azoles with immunosuppressive agents like tacrolimus, as azoles can interfere with CYP3A4 metabolism, increasing the risk of tacrolimus toxicity. Isavuconazole demonstrates the least interaction with immunosuppressants (Kozuch et al., 2024). Thus, managing these drug-drug interactions requires therapeutic drug monitoring (TDM) of both the azole and tacrolimus, with reduced tacrolimus doses to maintain blood concentrations within the therapeutic range (Berge et al., 2009). Given our *in vitro* and *in vivo* findings, the antifungal potentiation of PCZ by tacrolimus highlights PCZ as a promising candidate for IPA prophylaxis or treatment in SOT recipients on tacrolimus.

Moreover, PCZ has the advantage of exhibiting relatively low MICs against azole-resistant *A. fumigatus*, typically ranging from 0.5 to 2 mg/L (with a resistance breakpoint of > 0.25 mg/L) (European Committee on Antimicrobial Susceptibility Testing,; Breakpoint tables for interpretation of MICs for antifungal agents, version 10.0, 2020). This is significantly lower compared to itraconazole, especially in TR_34_/L98H mutants, where MICs often exceed 8 mg/L (European Committee on Antimicrobial Susceptibility Testing,). Similarly, TR_46_/Y121F/T289A mutants often demonstrate high resistance to VCZ and isavuconazole (resistance breakpoints > 1 and 2 mg/L respectively), with MICs occasionally exceeding 8 mg/L (European Committee on Antimicrobial Susceptibility Testing,; Breakpoint tables for interpretation of MICs for antifungal agents, version 10.0, 2020). Given the lower MICs of PCZ against azole-resistant isolates, even a modest increase in its concentration or efficacy could have clinical benefits. Strategies such as potentiation with tacrolimus may raise drug concentrations above the resistant isolate’s MIC, thereby overcoming resistance. Indeed, we showed *in vitro* and in *G. mellonella* that the combination of tacrolimus and PCZ exhibited antifungal activity against resistant *A. fumigatus*, while PCZ alone did not. Clinically, PCZ prophylaxis in patients on tacrolimus may therefore be beneficial against both azole-sensitive and -resistant *A. fumigatus* isolates, whereas other azoles may only target sensitive strains.

In our case report, which dates back to the pre-isavuconazole era, PCZ was preferred for prophylaxis over VCZ due to its more manageable interaction profile with the patient’s concomitant medications, as VCZ is a strong CYP3A4 inhibitor, a moderate CYP2C19 inhibitor and a weak CYP2C9 inhibitor, while PCZ only inhibits CYP3A4 and P-glycoprotein (Lempers et al., 2016). This case highlights the need to further examine PCZ prophylaxis in patients with native CF lungs, characterized by biofilms and chronic *A. fumigatus* airway infection, to prevent IPA development after SOT requiring calcineurin-inhibitors like tacrolimus. In a broader context, similar outcomes were observed in SOT recipients with cryptococcosis, where those on calcineurin inhibitors showed improved response to antifungal therapy with amphotericin B or fluconazole, and calcineurin inhibitor use was independently associated with lower mortality (Singh et al., 2007). In another case, a lung transplant patient on tacrolimus experienced *A. fumigatus* dissemination from the lung to the ankle and adjacent bone. After failing itraconazole and lipid amphotericin B therapies, the patient showed marked clinical improvement and no recurrence of infection with PCZ treatment (Lodge et al., 2004). Although no definitive conclusions on azole potentiation by tacrolimus can be drawn from these cases, this data provides a promising basis for further research on the clinical implications of azole therapy in SOT cohorts receiving tacrolimus.

To further develop this antifungal potentiation strategy, understanding the mechanism of action would help. Both tacrolimus and rapamycin are inhibitors of intracellular calcineurin in human and fungal cells and they both showed *in vitro* synergy with PCZ, thereby suggesting involvement of the calcineurin pathway in this antifungal synergy. In *A. fumigatus* and other fungal pathogens, the calcineurin pathway helps to protect the cell wall and membrane from antifungal damage, allowing fungi to resist antifungal stress. PCZ, which disrupts the fungal membrane by inhibiting ergosterol synthesis, may have its fungistatic activity converted to fungicidal when calcineurin is also inhibited, enhancing the overall antifungal effect (Shirazi and Kontoyiannis, 2013; Gao and Sun, 2015). Vice versa, membrane disruption by azoles can increase intracellular tacrolimus uptake, leading to cell death (Uppuluri et al., 2008). As such, the calcineurin pathway was shown to be directly involved in the synergy between tacrolimus or cyclosporine A and fluconazole against *C. albicans* biofilms (Uppuluri et al., 2008). In addition, tacrolimus is a known fungal efflux pump inhibitor, leading to enhanced intracellular accumulation of azoles (Del Poeta et al., 2000; Schwarz and Dannaoui, 2020). Alternatively, Wang et al. linked the synergistic effect between tacrolimus and azoles in *S. apiospermum* to increased ROS activity and apoptosis (Wang et al., 2022). Why some azoles interact synergistically with tacrolimus and others do not, as seen with PCZ and VCZ in our *in vitro* results, remains unknown. Further in-depth studies could establish the molecular mechanism of the synergy between tacrolimus and PCZ against *A. fumigatus* and other fungi.

To expand the application potential of tacrolimus in antifungal therapy, tacrolimus analogs with higher specificity for fungal calcineurin and minimal immunosuppressive effects on human cells have been developed. These analogs retain their antifungal potentiation properties without the immunosuppressive side effects (Nambu et al., 2017; Lee et al., 2018). As such, the tacrolimus analog 9-deoxo-31-*O*-demethyl-FK506, administered at 3 mg/kg, significantly improved survival in *C. neoformans-*infected mice when combined with fluconazole (Lee et al., 2018). These analogs represent a promising strategy to exploit the interaction between azoles and calcineurin-inhibitors in a broader, immunocompetent, patient population.

While this study provides important novel insights on the interaction between PCZ and tacrolimus, several limitations should be considered. The biofilm model we used is relatively simple, and more clinically relevant insights could be obtained by exploring more complex biofilm models that mimic the *in vivo* environment better (e.g. using lung epithelial cells). While the tacrolimus dose tested in *G. mellonella* was within the therapeutic dosing range, this model is not suitable to investigate complex pharmacokinetics. Moreover, *G. mellonella* larvae lack an adaptive immune system, particularly relevant regarding tacrolimus’s T-cell-inhibiting effect in humans. Therefore, it is essential to validate the synergy between tacrolimus and PCZ in a murine model of IPA including tacrolimus immunosuppression resulting in human therapeutic blood levels (typically 5-15 ng/mL). Clinical evidence for the advantage of PCZ over other azoles in patients receiving tacrolimus could be obtained through retrospective SOT cohort analyses and ultimately, randomized controlled trials in SOT patients on tacrolimus will be necessary to assess the prophylactic and therapeutic potential of PCZ compared to the standard of care in clinical settings.

In conclusion, a liver-transplant CF patient on tacrolimus showed remarkable eradication of chronic *A. fumigatus* colonization after PCZ treatment, prompting a preclinical investigation of PCZ-tacrolimus synergy. We found that immunosuppressants like tacrolimus and rapamycin enhance PCZ’s *in vitro* activity against both azole-sensitive and -resistant planktonic *A. fumigatus* isolates and against biofilm formation of azole-sensitive isolates. VCZ showed synergy only with rapamycin and only against biofilm formation of azole-sensitive *A. fumigatus. In vivo*, tacrolimus enhanced PCZ’s antifungal effect in a *G. mellonella* aspergillosis model. Together with our case report, these novel *in vitro* and *in vivo* findings highlight potential benefits of using PCZ in SOT recipients on tacrolimus. Further studies could unravel the underlying mechanism of this synergy, while additional murine and clinical investigations are warranted to fully evaluate the therapeutic benefits of PCZ versus VCZ in the patient population on tacrolimus.

## Data Availability

The original contributions presented in the study are included in the article/**Supplementary Material**. Further inquiries can be directed to the corresponding author.
