# Supplementary material for: Combinations of posaconazole and tacrolimus are effective against infections with azole-resistant Aspergillus fumigatus
Source: Front Cell Infect Microbiol. 2025 Apr 25;15:1550457. doi: 10.3389/fcimb.2025.1550457 (PMC12062170; doi:10.3389/fcimb.2025.1550457)
Supplement: Supplementary file 1 [file DataSheet1.docx]

**Supplementary data**

**Detailed case study: Posaconazole prophylaxis in cystic fibrosis patient with chronic pulmonary *Aspergillus* colonization in combination with immunosuppressant therapy after liver transplantation results in *Aspergillus* negative cultures**

In people with cystic fibrosis (CF), lung disease involves poor mucus clearance, leading to a vicious cycle of infection by micro-organisms forming biofilms at the epithelial-cell surface, inflammation, and further airway destruction (19). A 33-year-old man with CF, homozygous for the F508del *CFTR* mutation, was listed for liver transplantation due to severe intrahepatic cholangiopathy with recurrent cholangitis. Concomitant lung transplantation was not advised as pulmonary function was only moderately compromised (FEV_1_ of 60% predicted). In the year before transplant, sputum cultures were variably positive for Gram-negative bacteria (*Serratia marcescens*, *Acinetobacter calcoaceticus/baumanii* complex, *Escherichia coli* and *Haemophilus* spp.), and he was considered chronically colonized by methicillin-sensitive *Staphylococcus aureus* and *Aspergillus fumigatus* for over 14 years, with 6 in 9 *Aspergillus* positive respiratory samples over the last year. To prevent the development of invasive aspergillosis in the immediate post-transplant setting, the patient was started on posaconazole (PCZ) prophylaxis (300 mg twice on day 0, then 300 mg daily until day 42 post-transplant), besides broad-spectrum antibiotics (piperacillin-tazobactam 4 g four times a day during 14 days). PCZ was preferred over voriconazole (VCZ) for its more favorable interaction profile. As immunosuppressants, he received tacrolimus, mycophenolate and methylprednisolone. Strikingly, apart from one bronchoalveolar lavage fluid, cultures remained negative for *A. fumigatus* during 3 months post-transplant, with clear improvement of chronic respiratory symptoms during PCZ therapy despite severe immunosuppression. The unexpected clearance of *A. fumigatus* from native CF lungs after 14 years of chronic colonization immediately post-liver transplant raised the question whether PCZ’s antifungal activity against *A. fumigatus* biofilms was potentiated by tacrolimus.


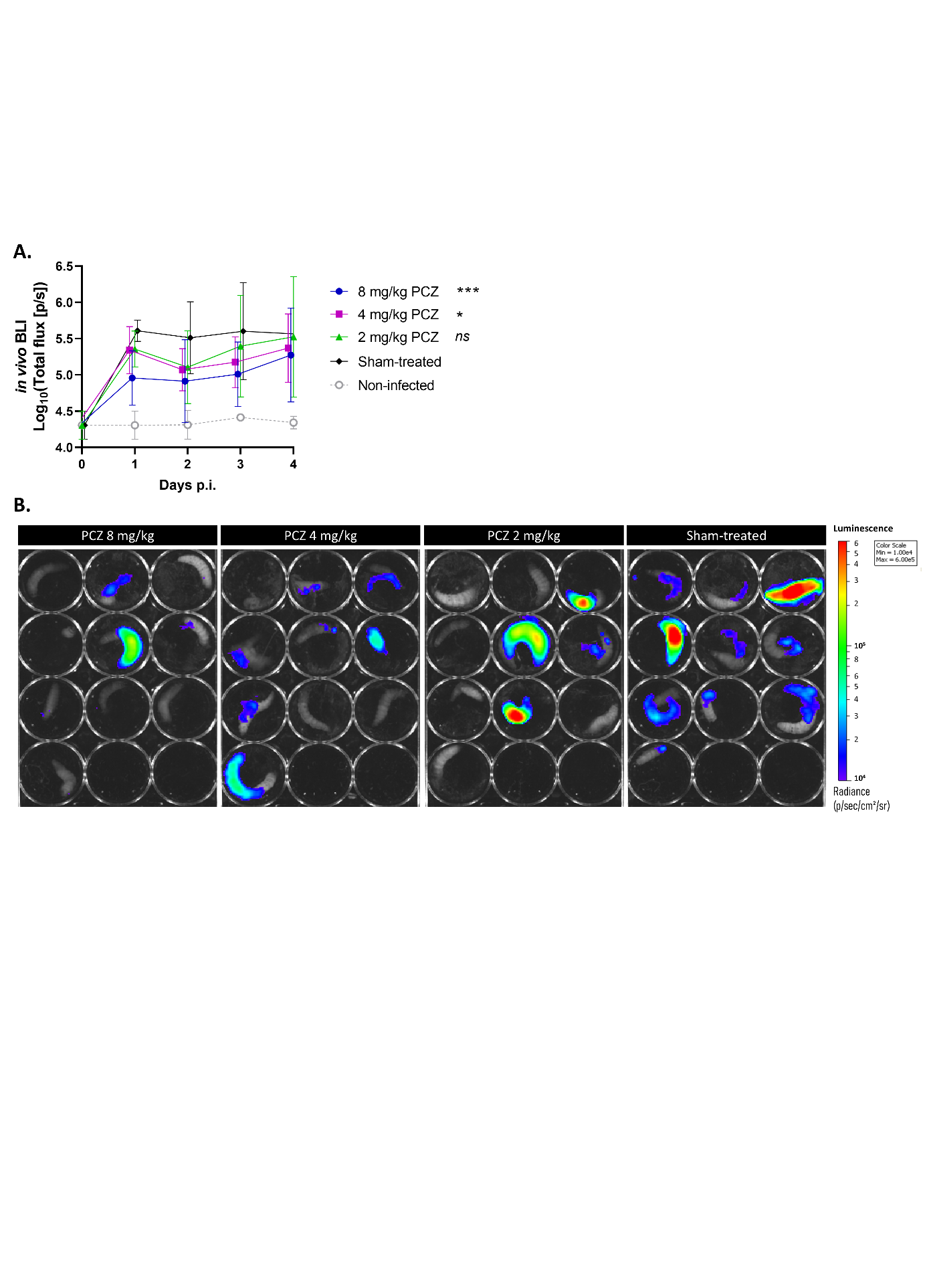


**Figure S1. Posaconazole dose titration against azole-susceptible *A. fumigatus* in *G. mellonella*. (A)** Antifungal treatment effect of different posaconazole (PCZ) doses on azole-susceptible *A. fumigatus* fungal burden in *G. mellonella* larvae measured by longitudinal *in vivo* BLI signal. Graphs show means ± standard deviations (SD) (*n* = 10). [p/s] = photons per second. Significance levels are compared to sham-treated: *, *P* < 0.05; **, *P* < 0.01; ns, non-significant. **(B)** Visual representation of *in vivo* BLI signal in infected larvae from panel A on day 3 p.i.

**
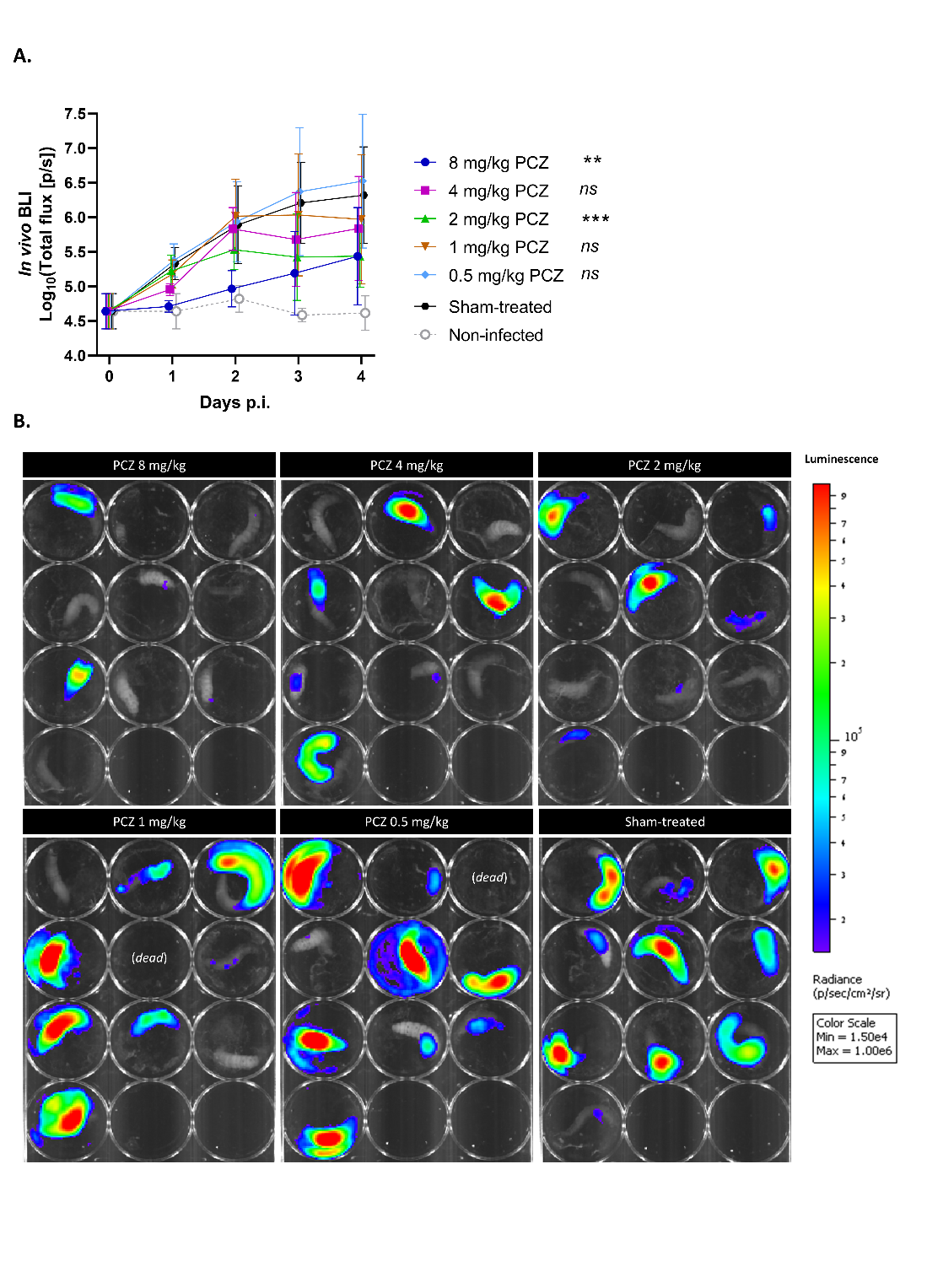
**

**Figure S2. Posaconazole dose titration against azole-resistant *A. fumigatus* in *G. mellonella.* (A)** Antifungal treatment effect of different posaconazole (PCZ) doses on azole-resistant *A. fumigatus* fungal burden in *G. mellonella* larvae measured by longitudinal *in vivo* BLI signal. Graphs show means ± standard deviations (SD) (*n* = 10). [p/s] = photons per second. Significance levels are compared to sham-treated: ***, *P* < 0.001; ****, *P* <0.0001; ns, non-significant. **(B)** Visual representation of *in vivo* BLI signal in infected larvae from panel A on day 3 p.i.

**
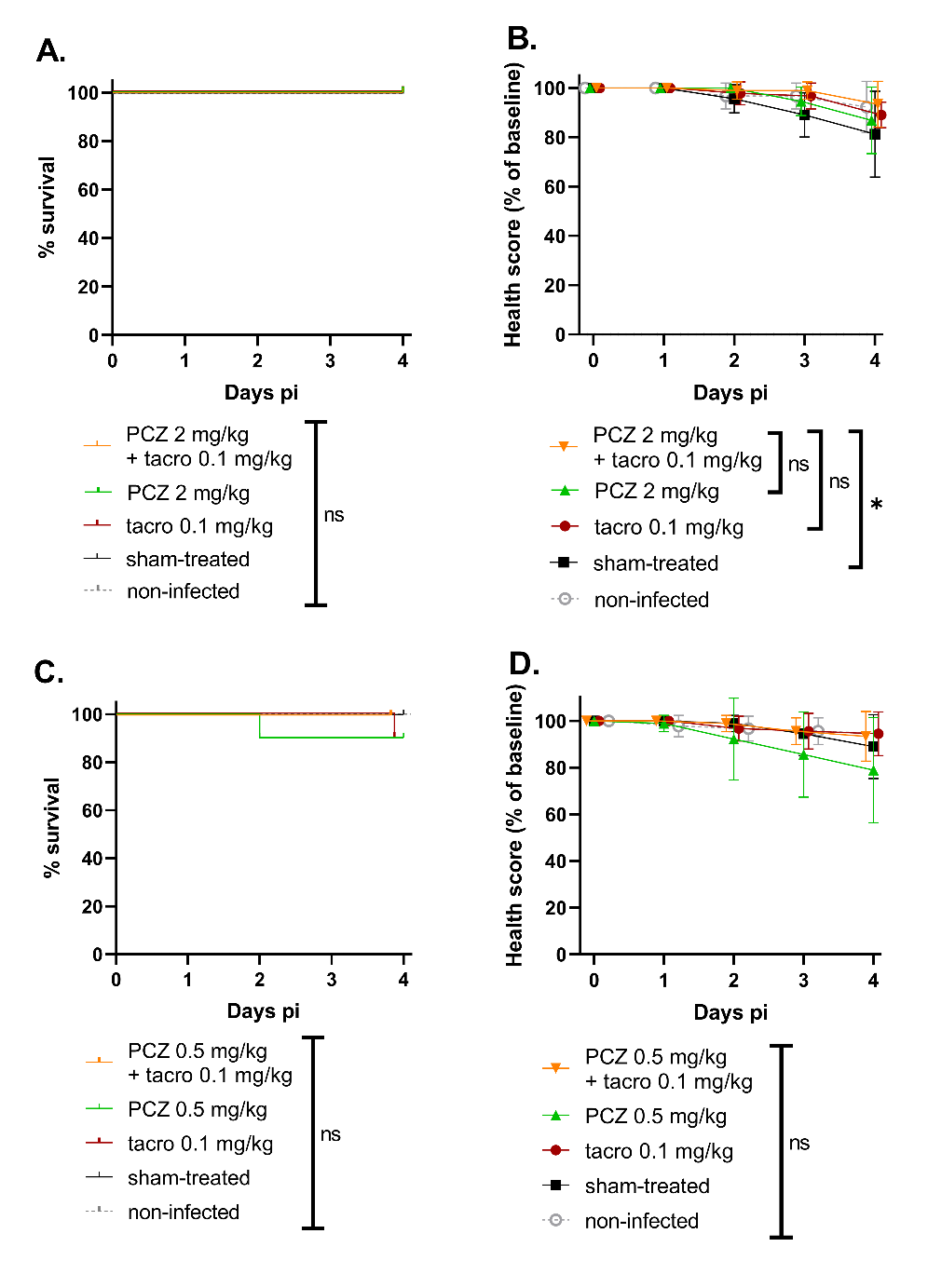
**

**Figure S3.** **Health readouts show good tolerability of posaconazole and tacrolimus by *G. mellonella* but are not sensitive enough to detect potentiation effects. (A,B)** Treatment effects of posaconazole (PCZ) and/or tacrolimus (tacro) on survival **(A)** and health scores **(B)** of *G. mellonella* larvae infected with azole-sensitive *A. fumigatus* over 4 days post infection (pi). **(C,D)** Treatment effect in larvae infected with the azole-resistant *A. fumigatus* (TR_34_/L98H) on survival **(C)** and health scores **(D)**. Health scores are mean (± SD) (*n*= 10). *, *P* < 0.05; ns, non-significant.
